# Characterization of SnO_2_-based ^68^Ge/^68^Ga generators and ^68^Ga-DOTATATE preparations: radionuclide purity, radiochemical yield and long-term constancy

**DOI:** 10.1186/s13550-014-0036-4

**Published:** 2014-07-24

**Authors:** Ferdinand Sudbrock, Thomas Fischer, Beate Zimmermanns, Mehrab Guliyev, Markus Dietlein, Alexander Drzezga, Klaus Schomäcker

**Affiliations:** Department of Nuclear Medicine, University Hospital of Cologne, Kerpener Str 62, Cologne, NRW 50937 Germany

**Keywords:** 68Ge-68Ga radionuclide generator, Radionuclide purity, Gamma-ray spectrometry, PET

## Abstract

**Background:**

With the increasing utilization of ^68^Ge-^68^Ga radionuclide generators, ^68^Ga labelled peptides like DOTATATE are receiving more attention in nuclear medicine. On the one hand, the long half-life of the parent nuclide ^68^Ge is an enormous advantage for routine applications, but the question of the long-term stability of the ^68^Ge breakthrough arises, which up to now has scarcely been investigated.

**Method:**

A sum of 123 eluates from four different ^68^Ge-^68^Ga generators (iThemba Labs, Faure, South Africa) and 115 samples of the prepared radiopharmaceutical ^68^Ga-DOTATATE were measured first with a dose calibrator and again after decay of the eluted ^68^Ga via gamma-ray spectrometry. A complete decay curve was recorded for one sample eluate. A further three eluates were eluted in ten fractions of 0.5 ml in order to obtain detailed information concerning the distribution of the two nuclides within the eluates. The influences of factors such as the amount of DOTATATE, addition of Fe^3+^ salts and replacement of HEPES buffer with sodium acetate on the radiochemical synthesis were also tested.

**Results:**

The content of long-lived ^68^Ge breakthrough increases over the entire period of use to more than 100 ppm. The labelling process with the chelator DOTA removes ^68^Ge efficiently. The maximum activity found in the residues of the radiopharmaceuticals investigated in this study was below 10 Bq in nearly all cases. In many cases (12% of the labelled substance), the long-lived parent nuclide could not be identified at all. The labelling process is still viable for reduced amounts of the chelator and with acetate buffer.

**Conclusion:**

Effective doses received by the patient from ^68^Ge in the injected radiopharmaceutical ^68^Ga-DOTATATE are lower than 0.1 μSv and are therefore practically negligible, especially when compared with the contribution of the PET radiopharmaceutical itself. Gamma-ray spectrometry as recommended by the European Pharmacopeia is suitable for quantification of radionuclidic impurities.

**Electronic supplementary material:**

The online version of this article (doi:10.1186/s13550-014-0036-4) contains supplementary material, which is available to authorized users.

## Background

In recent years, the generator-produced ^68^Ga has attracted increasing interest [1]–[8]. The ‘new trend’ [9] in nuclear medicine is based on the ^68^Ge/^68^Ga generator that has been discussed long [10]–[12]. Its short half-life makes ^68^Ga excellently suitable for imaging in nuclear medicine. Through trivalent ^68^Ga(III) cations bound to DOTA-conjugated peptides, the radiopharmaceutical gains high affinity to the somatostatin receptor SSTR2 [2],[6],[13],[14]. ^68^Ga undergoes disintegration via β^+^ decay and electron capture with a half-life of 67.8 min and emits high energy positrons of ca. 1.9 MeV (Table 1) [15].

**Table 1:** **Nuclear data for** ^**68**^ **Ga and** ^**68**^ **Ge**

Nuclide	Half-life	Gamma rays and abundance	Charged particles, particle type and abundance
Ga-68	67.629 min	805.75 keV (0.084), 1,077.35 keV (3.0), 1,260.97 keV (0.083), 1,883.09 keV (0.138)	821.7 keV*, β^+^ (1.2); 1,899.1 keV*, β^+^ (87.7); electron capture (11.1)
Ge-68	270.8 days	No gamma rays, determination via decay to Ga-68	Electron capture (100)

The abundance values are enclosed in parentheses. Asterisk denotes an endpoint energy.

The mother nuclide of ^68^Ga is the relatively long-lived ^68^Ge (*T*_1/2_ = 270 days) that nowadays is commonly produced by the nuclear reaction ^69^Ga(p,2n)^68^Ge on enriched ^69^Ga or on natural gallium as target material containing the stable nuclide ^69^Ga with an abundance of 60% [6]. The comparably long half-life of ^68^Ge gives ^68^Ga an enormous advantage as a generator-based radionuclide. Due to high reaction cross-sections in the energy range of 10 to 20 MeV [8], this reaction appeared suitable in spite of the long half-life of the product nuclide which usually requires a long irradiation. Details on the production, i.e. target materials, yields of different nuclear reactions for gallium targets and possible generators were studied in the early 1980s, e.g. [11],[16]. Meanwhile, different generator types have become commercially available, and the respective generator concepts are discussed in detail by Rösch [12]. The most common generators use matrices of TiO_2_ or SnO_2_ from which Ga^3+^ is eluted with HCl while Ge^4+^ remains adsorbed. The generator used in this study is based on SnO_2_, which, in comparison to TiO_2_, requires a higher concentration of the eluant (0.6 M instead of 0.1 M HCl) and, due to the lower pH, a different labelling strategy. Further discussions have focused on matrix-derived metallic impurities, which might affect the labelling efficiency [17].

The problem of unnecessary patient exposure to radiation could arise from two sources: ^68^Ge breakthrough that is not removed in the labelling process and possibly imperfect labelling, leading to an insufficient yield and, hence, to free ^68^Ga in the radiopharmaceutical. As they have an influence on the labelling process, traces of non-radioactive metallic contaminants in the eluate have to be taken into account. The requirements on the quality of the primarily eluted gallium chloride solution as well as the labelled compound are defined in the European Pharmacopeia (Ph Eur) [18],[19]. The radionuclidic purity of ^68^Ga in the gallium chloride solution is limited to a minimum of 99.9% of the total radioactivity [18], but the content of ^68^Ge should not exceed 0.001% [18]. Gamma-ray spectrometry is recommended as the method of choice for the determination and quantification of the impurities. The European Pharmacopeia states precisely that peaks in the gamma-ray spectra that do not belong either to ^68^Ga (Table 1), to annihilation radiation or a possible sum peak at 1,022 keV shall ‘represent not more than 0.1 per cent of the total radioactivity’ [18].

In this study, we assessed the ^68^Ge breakthrough in 123 eluates of four SnO_2_-based ^68^Ge/^68^Ga generators and the ^68^Ge content in 115 samples of the radiopharmaceutical after preparation of ^68^Ga-DOTATATE in order to measure the depletion of ^68^Ge during a manual radiochemical synthesis. We studied the influence of different amounts of DOTATATE on the labelling process, the feasibility of different buffers (acetate instead of HEPES) as well as the influence of interfering ions such as Fe^3+^.

## Methods

### Generators, elution procedure and radiochromatography

Studies were performed using four generators between December 2010 and April 2013. All generators had a nominal activity of 1,850 MBq and were supplied by iThemba Labs (Faure, South Africa provided by IDB Holland BV, Baarle-Nassau, The Netherlands) [4],[20]. According to the manufacturer, the mother nuclide is produced via the nuclear reaction ^69^Ga(p,2n)^68^Ge. Apart from the ^68^Ge breakthrough, minor metallic impurities (Zn, Fe, Sn, Ti, Cu and Al) represent a problem that has to be taken into account [20]. As a decay product of the long-lived mother nuclide, the concentration ^68^Zn is likely to increase over the entire period of use (see, e.g. [17],[20] for details).

The first three generators were used over a period of 9 months, while the fourth generator was in use between February and October 2013. ^68^Ga is eluted from the generator with 5 ml HCl (*c* = 0.6 M) [20].

Three samples of ^68^Ga eluates were measured repeatedly over a period of up to 7 months in order to obtain detailed decay curves. A dose calibrator (VDC405, Veenstra Instruments, Joure, The Netherlands) was used for the determination of ^68^Ga activities directly after elution (first day), and measurements of the activity were continued, after complete decay of the primarily eluted ^68^Ga, by high-resolution gamma-ray spectrometry using a high-purity germanium detector system (HPGe, Ortec, Oak Ridge, TN, USA).

Radiochromatography was performed by a fractionated elution of the generator for three illustrative samples. In this case, the generator was eluted as usual with 5 ml HCl (0.6 M), and the eluate was separated into ten fractions of 0.5 ml each. Each fraction was measured first using the dose calibrator and again, after decay of the primarily eluted ^68^Ga, using gamma-ray spectrometry.

### Standard manual radiochemical preparation of ^68^Ga-DOTATATE

A 500 mg (±3 mg) HEPES (Sigma-Aldrich Chemie GmbH, Taufkirchen, Germany) was transferred into a sterile 10-ml glass vial. A 50 μg DOTATATE (JPT, Berlin, Germany) was dissolved in 100 μl Ultrapur® water (Merck Millipore, Darmstadt, Germany) and added to the dry HEPES powder. The ^68^Ge/^68^Ga-generator was eluted with 5 ml of 0.6 M HCl (made from 30% HCl, and water, both Ultrapur® grade, Merck Millipore). Two milliliters of the ^68^Ga containing eluate was transferred to the HEPES/DOTATATE mixture. After very careful shaking, the mixture was heated for 15 min at 85°C in a heating block. It was then removed from the heating block and cooled down for 10 min before being diluted with 6 ml water (Ultrapur®). The labelling mixture was purified on a SepPak light C18 cartridge (Waters, Eschborn, Germany) with 10 ml water (Ultrapur®) and eluted with 2 ml ethanol (70%, made from ethanol, ROTIPURAN®, ROTH, Karlsruhe, Germany, and Ultrapur® water). The cartridge was pre-conditioned by rinsing first with 5 ml ethanol (70%), then with 10 ml Ultrapur® water and 5 ml air. Four milliliters of sterile isotonic saline (BERLIN-CHEMIE AG, Berlin, Germany) was added to the ethanolic solution for injection. The preparations were followed by a sterile filtration, and before administration, the product is diluted with approximately 20 ml saline.

The radiochemical yield of the manual syntheses was expressed as percentage of the activity used for the preparation of ^68^Ga-DOTATATE and the activity in the final product. The time for the whole process was exactly recorded (typically 30 min), and therefore, the radioactive decay in the meantime could be corrected for.

### The robustness of the manual radiochemical preparation tested under different conditions

The robustness of the labelling process was tested to establish three points:

- What is the critical amount of DOTATATE?
- Can HEPES be replaced by acetate as buffer?
- What is the critical concentration of iron as ubiquitous metallic impurity?

#### Different amounts of DOTATATE and acetate buffer

In order to analyse parameters that may influence the abovementioned standard preparation technique, the synthesis was carried out with three modifications for test purposes. Thirteen preparations were performed with different amounts of DOTATATE (1, 3, 7, 10 and 15 μg) and three further preparations with sodium acetate (130 and 164 mg) instead of HEPES buffer.

#### Addition of Fe^3+^

A further eight preparations were carried out after the addition of Fe(NO_3_)_3_ · 9 H_2_O (Sigma-Aldrich) with different volumes and concentrations to obtain different molar amounts of the trivalent cation. Fe(NO_3_)_3_ · 9 H_2_O was added in the following concentrations and volumes: 1 mmol/l (100 and 10 μl), 500 μmol/l (10 μl), 100 μmol/l (10 μl), 50 μmol/l (10 μl), 5 μmol/l (10 μl) and 1 μmol/l (100 and 10 μl twice). Amounts of between 10^−11^ and 10^−7^ moles of Fe(NO_3_)_3_ · 9H_2_O were thereby obtained.

The radiochemical yields of the syntheses were assessed for all tests. The radiochemical yield was expressed as the fraction of the activity measured in the radiopharmaceutical in relation to the activity of ^68^Ga added to DOTATATE after decay correction for the elapsed time. Radiochemical purity was routinely assessed via thin-layer chromatography (TLC, ITLC-SG, citrate buffer 0.1 M).

### Samples, dose calibrator, gamma-ray spectrometry and nuclear data

**Figure 1:**
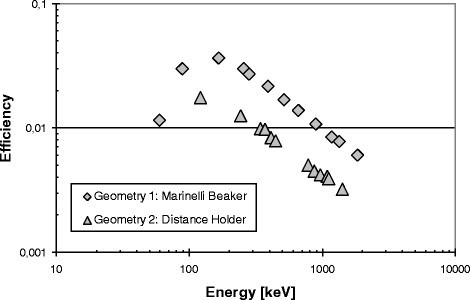
**Efficiency of the high-purity germanium detector.**

^68^Ga was measured with a dose calibrator VDC-405 in the eluates immediately after elution and in the radiopharmaceutical immediately after preparation. The activities of ^68^Ge in aliquots of eluates and radiopharmaceuticals were determined after complete decay of ^68^Ga (*t* > 1 day) via gamma-ray spectrometry using an electrically cooled ‘poptop’ coaxial HPGe detector with a crystal diameter and length of 64.9 and 54.6 mm, respectively. The detector has a high energy resolution of 0.95 keV @ 122 keV ^57^Co and 1.9 keV @ 1,332 keV ^60^Co. The counting efficiency for two positions over the whole energy range from 50 to 2,000 keV was determined using calibrated standard samples (Figure 1). Spectral analyses were performed by use of the programme Gamma-W (Dr. Westmeier GmbH, Ebsdorfergrund-Mölln, Germany).

Over the study period, a total of 218 manual radiochemical and a further 13 test preparations of ^68^Ga-DOTATATE were performed. The content of ^68^Ge was determined in 123 eluates and 115 different ^68^Ga-DOTATATE samples.

^68^Ge was determined in the eluates and the ^68^Ga-DOTATATE residues using the 1,077-keV gamma-ray emission of the daughter nuclide ^68^Ga, assuming that ^68^Ga is replenished through decay of a possible breakthrough of germanium and therefore detectable (Table 1).

### Extremity dosimetry of the personnel

A total of 38 preparations over a period of 6 months were monitored by means of a finger ring dosimeter (position: middle finger, right hand) that was worn and evaluated monthly by the North Rhine-Westphalian Measurement Office in Dortmund (Materialprüfungsamt Dortmund) in accordance with German legislation. The number of preparations was recorded for every month. The dose quantity recorded for measurements of the hand was the personal dose equivalent H_*p*_(0.07).

## Results and discussion

### Results

#### Radiochromatography

**Figure 2:**
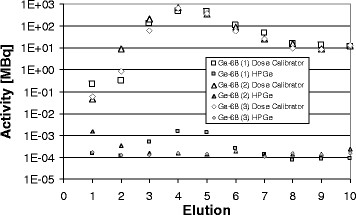
**Three elution profiles for** ^**68**^ **Ga (large symbols) and** ^**68**^ **Ge (small symbols).**

Radiochromatography was performed on three eluates in which a total of 1,221, 1,255 and 1,407 MBq ^68^Ga was eluted in ten fractions of 0.5 ml. Between 1,100 and 1,300 MBq ^68^Ga (93% to 95%) were obtained in fractions 3 to 6 (Figure 2). Only two fractions alone (nos. 4 and 5) contain more than 70% of the total eluted activity. These two fractions represent a very high activity concentration.

The sum of the ^68^Ge breakthrough in these ten fractions yielded between 1.4 and 4.1 kBq. Hence, the fraction of long-lived ^68^Ge in the ^68^Ga eluate corresponded to 1.2 to 3.3 ppm (0.00012% to 0.00033% or 1.2 to 3.3 Bq (^68^Ge)/1 MBq (^68^Ga)).

#### Gamma-ray spectrometry, decay curves and radionuclide impurities

**Figure 3:**
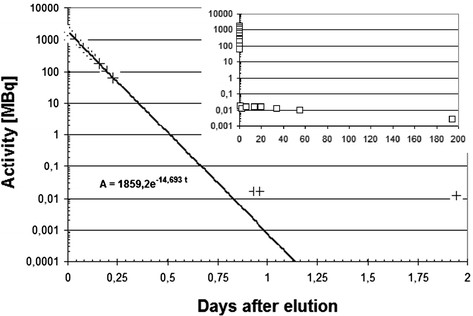
**Decay curve for one eluate measured over 2 days.** Inset: complete decay curve over 200 days.

All eluates were measured immediately after elution using the dose calibrator and later on by means of gamma-ray spectrometry. A complete decay curve of one illustrative eluate is displayed in Figure 3. One day after elution, the activity in the eluate is ten times higher than would be expected from the calculated decay curve which is caused by a breakthrough of ^68^Ge. Accordingly, the complex decay curve exhibits two different Half-lifes: for the first day after elution, a half-life of 67.93 min is found (dose calibrator), while from day 2 to day 200, the calculated half-life is 283 days (HPGe). The physical half-lives are well reproduced for ^68^Ga and acceptably well for ^68^Ge (Figure 3).

**Figure 4:**
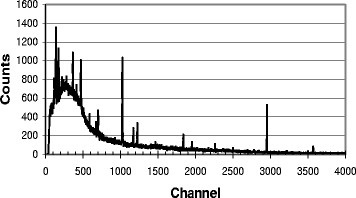
**Gamma-ray spectrum of** ^**68**^ **Ga-DOTATATE.**

**Figure 5:**
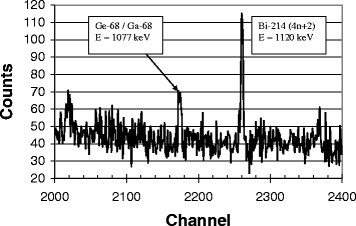
**Gamma-ray spectrum of**^**68**^**Ga-DOTATATE.** Detailed view of the region around channel no. 2200 (1,000 to 1,200 keV).

In the gamma-ray spectra of all eluates, peaks attributable to ^68^Ga could be clearly identified a few days after elution. The branching ratios of the gamma-ray emissions of ^68^Ga are small (<5%, Table 1), but peaks belonging to the four most abundant transitions were all found within the spectra and none of them was obscured by any peak from other nuclides, which would lead to an alteration of the full-width at half maximum (FWHM) of the respective photopeak. After a decay time of 2 days, further observable peaks could be assigned to nuclides from the natural decay chains or ^40^ K, the latter nuclide with a peak around channel no. 2900 (Figures 4 and 5).

The activity of ^68^Ge in ^68^Ga-DOTATATE residues with activities in the range between 10 and 30 MBq ^68^Ga was in nearly all cases below 10 Bq of ^68^Ge. For a measuring time of 1 to 3 days, the peak areas yielded hundred counts or even fewer, and in some cases (*N* = 14), the peak region around 1,077 keV was completely indistinguishable from the background. Yet, a few samples measured over more than 8 days undoubtedly gave a peak at 1,077 keV, albeit a ‘weak’ or ill-pronounced peak after 2 or 3 days. It was thus not practicable to measure many more than 100 reserve samples for 8 days or longer. Due to fluctuation of the background, it was also not feasible to record and subtract a constant background activity for each sample so that the activities of the given impurities in ^68^Ga-DOTATATE preparations strictly represent an upper limit for the content of long-lived impurities. For the 1,077 keV photopeaks in the spectra, the background subtraction routine of the programme Gamma-W was applied.

A typical gamma-ray spectrum of a residue of ^68^Ga-DOTATATE is shown in Figure 4, and a partial view of the region around 1,077 keV is shown in Figure 5. The peak at 1,120 keV (naturally occurring radionuclide from the decay chain 4*n* + 2) can be seen more clearly. In ^68^Ga-DOTATATE samples, the fraction of ^68^Ge in the radiopharmaceutical is, on average, below 1 ppm (<0.0001%, mean 0.7 ppm, median 0.3 ppm). No further peaks could be observed (Figures 4 and 5), and hence, gamma-spectrometry proves that in all samples, only ^68^Ge has to be considered as radionuclidic impurity.

Typically less than 10 Bq of ^68^Ge was detected in each single residue of ^68^Ga-DOTATATE (*N* = 101 of 115) via gamma-ray spectrometry [21]. In many residues, the long-lived mother nuclide could not be identified at all (*N* = 14 of 115). Sample descriptions, i.e. activities of the residues of the eluates and DOTATATE are summarized in Table 2.

**Table 2:** **Statistical distribution of activities in the** ^**68**^ **Ga eluates and in** ^**68**^ **Ga-DOTATATE**

Sample		Activities (Ga-68)	Activities (Ge-68)
Eluate (*N* = 123)	Max	1,500 MBq	776 kBq
	Min	41.5 MBq	0.1 kBq
	Mean	474 MBq	50 kBq
	Median	376 MBq	27 kBq
DOTATATE (*N* = 115)	Max	68.2 MBq	11.4 Bq
	Min	0.42 MBq	0 Bq*
	Mean	21.1 MBq	4.6 Bq
	Median	20.7 MBq	4.3 Bq

Asterisk means not detectable.

**Figure 6:**
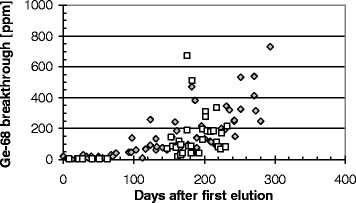
**Long-term control of the** ^**68**^ **Ge breakthrough in two generators (□ = generator 2, ◊ = generator 3).**

The breakthrough in the eluates increases significantly over the shelf-life of the generator as shown in the example in Figure 6. In the first 25 samples eluted within the first 50 days, the ^68^Ge activities remain low (<1 kBq) but then increase. After a few months, the breakthrough is usually in the region of 100 to 300 ppm (0.01% to 0.03%) with three single outliers beyond 500 ppm (0.05%). The breakthrough of ^68^Ge shows a maximum of 800 ppm (0.08%) (Figure 6). The long-term constancy for generator no. 2 was comparable to that for generator no. 3, and the first 25 elutions of generator no. 4 show similar results. Taking into account that ^68^Ge is presumably the only radionuclidic impurity, the requirement of the European Pharmacopeia for ^68^Ga (99.9%) is therefore ensured.

#### The robustness of the radiochemical preparation tested under different conditions

**Figure 7:**
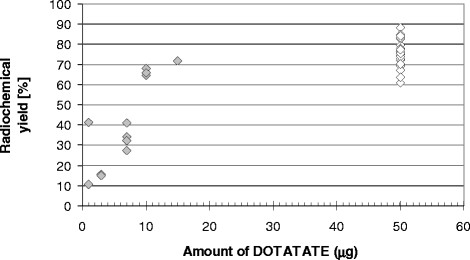
**Radiochemical yield (**^**68**^**Ga-DOTATATE) for syntheses with different amounts of DOTATATE (filled diamonds).** Yield of 20 routine preparations (empty diamonds).

The radiochemical purity of the purified injected product was, in all cases, 99% (TLC). The radiochemical yield (activity in the labelled product in relation to the initial activity of ^68^Ga) ranges from 60% to 90% (Figure 7). Figure 7 shows the yield of 20 routine preparations carried out at the same time when the modified syntheses were tested.

##### Different amounts of DOTATATE and acetate buffer for manual labelling

After the addition of 1 μg DOTATATE, the radiochemical yield of the preparation was 11% and 41%, while 3 μg DOTATATE yielded 15% and 16%, and 7 μg DOTATATE yielded 27% and 41%. Four preparations with 10 μg DOTATATE yielded between 64% and 72%, whereas the yield after addition of 15 μg DOTATATE fell back to 43% (Figure 7). The radiochemical yield for a total of 20 routine preparations was found to lie between 60% and 90% (mean and median 75%). These 20 standard manual procedures were performed at the same time as the radiochemical test.

The content of ^68^Ge in the radiopharmaceutical ranged from a non-detectable breakthrough (two samples) to 34 Bq of ^68^Ge after addition of 3 μg DOTATATE. The values for 1 and 3 μg DOTATATE were slightly higher (11 to 34 Bq) than those for higher amounts of DOTATATE (7 to 19 Bq). The radiochemical yield with sodium acetate as buffer was found to lie between 74% and 82% (*N* = 3).

##### Addition of Fe^3+^

**Figure 8:**
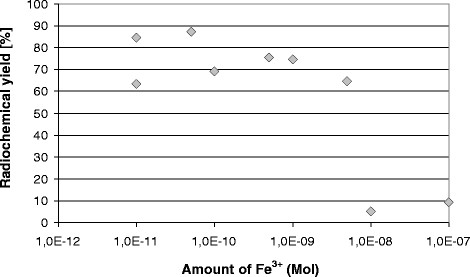
**Radiochemical yield (** ^**68**^ **Ga-DOTATATE) after addition of different molar amounts of Fe** ^**3+**^ **.**

For different concentration of Fe^3+^ cations, the yield was also 74% (mean value) in most cases (7 out of 9 preparations) (Figure 8). But after addition of higher amounts of the trivalent cation (10 or 100 μl of a solution with a molar concentration of 1 mM), the radiochemical yield decreased to 10% (Figure 8), and the activity of ^68^Ga-DOTATATE became too low, i.e. in the region of only 10 to 30 MBq. The ^68^Ge activity in the labelled ^68^Ga radiopharmaceutical remained in all cases below 5 Bq.

#### Extremity dosimetry of the personnel

The monthly assessed personal dose equivalent H_*p*_(0.07) was found to lie between 0 mSv (one preparation per month in the recording period) and 5 mSv (ten preparations per month in the recording period). The average dose, i.e. the monthly personal dose divided by the number of preparations, varied between 0 and 0.7 mSv, while the average dose over the whole study period yielded 0.5 mSv for each synthesis.

### Discussion

#### Radiochromatography

When a fractionated elution is carried out, almost the complete amount of ^68^Ga is collected in four fractions (nos. 3 to 6) or within 1.5 to 3 ml hydrochloric acid (0.6 M), respectively. This is in good agreement with previous reports from authors who used the most common TiO_2_-based generator [15],[17],[22]–[25] and in agreement with a few authors who used a SnO_2_-based generator from iThemba Labs, too [20]. The elution profile presented in this work is comparable to TiO_2_-based generators, although these two generators are eluted with significantly different concentrations of hydrochloric acid. The principle form of the elution profile was described very early on by Pao et al. [16] for an elution via hydrous zirconium oxide.

#### Gamma-ray spectrometry, decay curves and radionuclide impurities

The breakthrough is specified by the manufacturers of the ^68^Ge/^68^Ga generator as 0.0035% or 35 ppm, which would be equivalent to 64.75 kBq in 1,850 MBq eluate. For the first elutions after delivery of the generator, the breakthrough does not in fact exceed this limit. But strictly speaking, the limit of 0.001% suggested by the European Pharmacopeia [18] is slightly exceeded in several eluates from the three generators evaluated in this study.

The half-life found for the decay of the eluate on the first day after elution (dose calibrator) reproduces the reported half-life of ^68^Ga very well, while later, the decay of the long-lived component follows with the half-life of ^68^Ge. This is a first indication of a high radionuclidic purity which is proven in detail for longer-lived nuclides via gamma-ray spectrometry carried out after decay of the primarily eluted ^68^Ga. Hence, as further radionuclidic impurities can largely be excluded, the requirements of the European Pharmacopeia concerning the radionuclide purity for ^68^Ga of 99.9% is most probably fulfilled [18],[19].

The reason for the variable but increasing breakthrough over time is not yet clear, but it does not appear to depend on the time between the two elutions. The time-dependent increase of the breakthrough resembles that found by Asti et al. [17], Lin et al. [24] and Loktionova et al. [26] for a different type of generator. For the generator used in this study, the breakthrough exceeds 100 ppm (10^−2^%) after a shelf life of 100 days, whereas the values presented by Lin et al. display a lower breakthrough, especially for one of the TiO_2_-based generators investigated by those authors [24].

In relation to the breakthrough of ^68^Ge in the eluate, the final product is depleted by more than a factor of 500 (Table 2). Compared to the results of Belosi et al. for the syntheses of ^68^Ga-DOTANOC [2], the preparation of ^68^Ga-DOTATATE as described in this study removes ^68^Ge relatively efficiently. Other authors cited by Belosi et al. [2] report an upper limit of 25 Bq ^68^Ge in ^68^Ga-DOTANOC. This is in good agreement with our findings (Table 2). The significant reduction of the ^68^Ge(IV) cations is presumably based on the fact that the complexation of cations with DOTA is most effective for trivalent metals [20],[27].

As previously mentioned by Loc’h et al. [11], it is difficult to detect traces of further nuclides in the eluate due to the high-energy emissions (Table 1) producing a high Compton background. Methods to reduce the Compton continua such as dedicated anti-Compton spectrometers would be needed to circumvent this problem [28]. But the presence of further nuclides in the radiopharmaceutical can be largely excluded with one principal exception: nuclides that do not emit gamma rays (pure β-emitters, EC, etc.), which cannot be detected by this method but might be present in the eluate and the radiopharmaceutical. But it was clearly confirmed for ^68^Ge that the preparation reduces Ge cations efficiently. Thin-layer chromatography is suitable for the detection of the majority radiochemical impurities, but it has been argued that some impurities formed by radiolysis may only be detected by HPLC [29].

#### The robustness of the radiochemical preparation tested under different conditions

Even the tiniest addition of 1 μg DOTATATE used in our tests represents an excess of the chelator (1 μg ~ 7 × 10^−10^ moles) in comparison to the amount of ^68^Ga (150 MBq ~ 10^−10^ g ~ 1.5 × 10^−12^ moles) and should therefore be sufficient for the preparation. The labelling efficiency should therefore be similar to higher DOTATATE amounts. However, this is obviously not the case and might be explained by the influence of metallic impurities. Nevertheless, the addition of only 1 to 7 μg DOTATATE is sufficient for removing the Germanium breakthrough.

For an addition of 10 μg or more, the yield of the labelling process was between 65% and 68%. The synthesis obviously remains robust down to this amount of DOTATATE and delivers a product sufficient for imaging even with an ‘aged’ generator.

Sodium acetate, an easily available and pharmacologically harmless buffer, is obviously able to replace HEPES. HEPES is a Good buffer for maintaining physiological conditions in cell cultures but poses some problems like long-term stability especially when exposed to humid air and light. Acetate as buffer represents further advantages to HEPES as it is more easily available and cheaper.

For DOTATATE amounts of 50 μg (3.5 × 10^−8^ moles), the addition of iron becomes critical in the presence of more than 10^−8^ moles of Fe^3+^. Any traces of iron as contaminant in solutions used for the radiochemical synthesis will easily exceed this limit and will hence impede the preparation of ^68^Ga-DOTATATE. One may draw the conclusion from the two ‘titration curves’ (Figures 7 and 8) that the metallic impurities are in the region of 7 × 10^−9^ to 7 × 10^−8^. Though the manual preparation is obviously robust, it remains questionable if manual syntheses do perfectly agree with today’s requirements of good manufacturing practice.

#### Dosimetric implications

##### Patients

The manual preparation of ^68^Ga-DOTATATE reduces the ^68^Ge content to less than 1 ppm, i.e. the application of 200 MBq ^68^Ga-DOTATATE to the patient leads to an unwanted incorporation of long-lived ^68^Ge in the region of 200 Bq or even less [2],[30]. A background subtraction would lead to an even further reduction of the calculated content of ^68^Ge in ^68^Ga-DOTATATE. Our finding of an almost negligible and barely detectable amount of ^68^Ge amount in ^68^Ga-DOTATATE components is therefore not inconsistent with authors who state that they did not find any ^68^Ge. Given an incorporated activity of 200 Bq ^68^Ge per application of 200 MBq ^68^Ga-DOTATATE, a minor total effective dose of 0.1 μSv [31] is certainly not exceeded. This dosimetric estimation supports the assessment of Breeman et al. [30].

##### Personnel

The extremity dose is an important quantity for the exposure of staff that has to be assessed regularly, especially for a manual preparation with high-energy beta emitters. Dosimetry by means of a finger ring dosimeter demonstrated that annual dose limits will not be exceeded. The data presented in this work represent an upper limit (0.5 mSv/synthesis) because other handlings of radioactivity apart from preparations with ^68^Ga are recorded as well. A high number of 200 preparations performed by one person will yield 100 mSv, and even when taking the position of the ring into account, which does not necessarily represent the highest extremity exposure, the annual limit of 500 mSv is unlikely to be reached.

## Conclusions

### Manual labelling

^68^Ga-DOTATATE can be prepared manually and safely by the method described in this study without using any dedicated automatic apparatus for synthesis. The synthesis is robust as it will not fail when using more than 10 μg of the chelator (2 ml eluate).

### Metallic impurities

Interfering trivalent ions like Fe^3+^ (>10^−8^ moles, i.e. 2 nmol/ml) need to be excluded. In this study, it was shown that the concentration of the interfering ions becomes critical in the region of 7 × 10^−9^ moles, i.e. 1.4 nmol/ml.

### Elution profile

If a high concentration of ^68^Ga in the eluate is required, it appears recommendable to discard the first 1.5 ml and to collect the following 1.5 ml for the radiochemical labelling.

### Breakthrough and radionuclidic purity

A small breakthrough of ^68^Ge into ^68^Ga is inevitable. The radionuclidic purity of ^68^Ga in the eluate meets the requirements of the European Pharmacopeia where a minimum of 99.9% ^68^Ga in gallium chloride solutions is specified. The ^68^Ge content exceeds the specification of 0.001% in some eluates, but in the labelled compound, it certainly does not exceed this limit.

### Gamma-ray spectrometry

Gamma-ray spectrometry as e.g. recommended by the Monograph of the European Pharmacopeia is suitable for the quantification of radionuclidic impurities in both the eluate and the labelled substance. As no other nuclides are present in the samples, scintillation counting would be sufficient, though high-resolution spectrometry using high-purity germanium detectors is superior.

### Dose estimation for the patient

The doses received by the patient from the long-lived breakthrough are in the region of 0.1 μSv.

### Radiation exposure of staff members

The manual labelling can be carried out without any critical extremity exposures.

## Authors’ information

FS is a nuclear chemist, radiochemist and a medical physics expert. TF is chemist and a specialist for radiopharmacy, BZ is a technologist. MG is a Ph.D. student. MD is a medical doctor. AD is a medical doctor and head of the Department of Nuclear Medicine, University Hospital of Cologne. KS is a radiochemist, radiobiologist and a qualified person in radiopharmacy. He is also the head of the radiopharmacy group, Department of Nuclear Medicine, University Hospital of Cologne.

## Authors’ original submitted files for images

Below are the links to the authors’ original submitted files for images. Authors’ original file for figure 1 Authors’ original file for figure 2 Authors’ original file for figure 3 Authors’ original file for figure 4 Authors’ original file for figure 5 Authors’ original file for figure 6 Authors’ original file for figure 7 Authors’ original file for figure 8
