# Supplementary figures and images for: Characterization of SnO2-based 68Ge/68Ga generators and 68Ga-DOTATATE preparations: radionuclide purity, radiochemical yield and long-term constancy
Source: EJNMMI Res. 2014 Jul 24;4:36. doi: 10.1186/s13550-014-0036-4 (PMC4884004; doi:10.1186/s13550-014-0036-4)

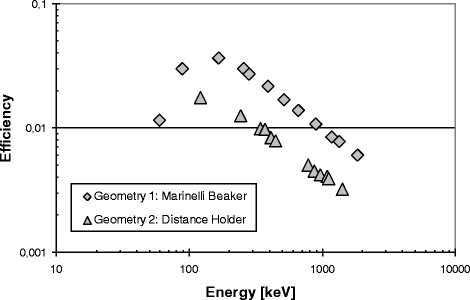

Supplement: Supplementary file 1 — Authors’ original file for figure 1 [file 13550_2014_36_MOESM1_ESM.gif]

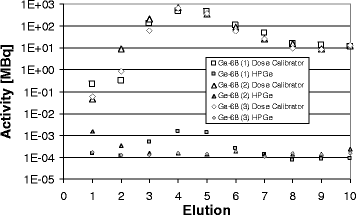

Supplement: Supplementary file 2 — Authors’ original file for figure 2 [file 13550_2014_36_MOESM2_ESM.gif]

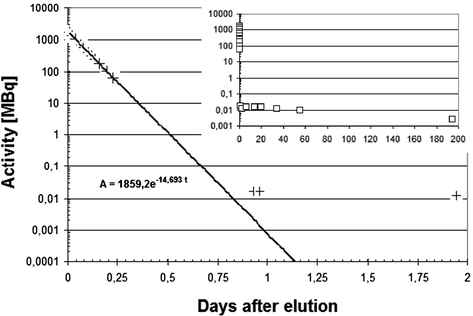

Supplement: Supplementary file 3 — Authors’ original file for figure 3 [file 13550_2014_36_MOESM3_ESM.gif]

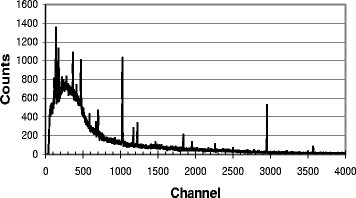

Supplement: Supplementary file 4 — Authors’ original file for figure 4 [file 13550_2014_36_MOESM4_ESM.gif]

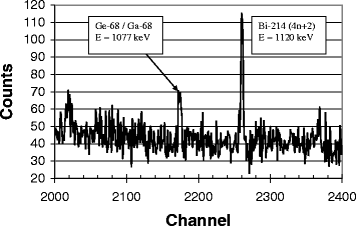

Supplement: Supplementary file 5 — Authors’ original file for figure 5 [file 13550_2014_36_MOESM5_ESM.gif]

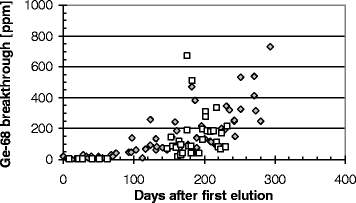

Supplement: Supplementary file 6 — Authors’ original file for figure 6 [file 13550_2014_36_MOESM6_ESM.gif]

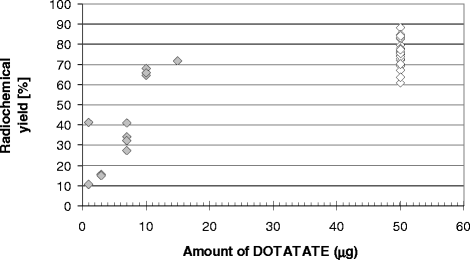

Supplement: Supplementary file 7 — Authors’ original file for figure 7 [file 13550_2014_36_MOESM7_ESM.gif]

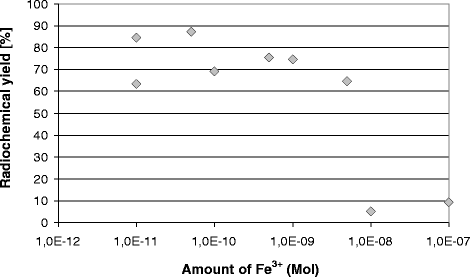

Supplement: Supplementary file 8 — Authors’ original file for figure 8 [file 13550_2014_36_MOESM8_ESM.gif]
